# Xpert bladder cancer monitor to predict the need for a second TURB (MoniTURB trial)

**DOI:** 10.1038/s41598-023-42088-z

**Published:** 2023-09-18

**Authors:** Johannes Breyer, Markus Eckstein, Danijel Sikic, Felix Wezel, Florian Roghmann, Mirco Brehmer, Ralph M. Wirtz, Jonas Jarczyk, Philipp Erben, Veronika Bahlinger, Franziska Goldschmidt, Guido Fechner, Jack Chen, Ellen Paxinos, Michael Bates, Maximilian Haas, Friedemann Zengerling, Christian Bolenz, Maximilian Burger, Arndt Hartmann, Maximilian C. Kriegmair, BRIDGE Consortium e.V., Johannes Breyer, Markus Eckstein, Danijel Sikic, Felix Wezel, Florian Roghmann, Ralph M. Wirtz, Jonas Jarczyk, Philipp Erben, Veronika Bahlinger, Franziska Goldschmidt, Guido Fechner, Maximilian Haas, Friedemann Zengerling, Christian Bolenz, Maximilian Burger, Arndt Hartmann, Maximilian C. Kriegmair

**Affiliations:** 1 Department of Urology, Caritas St. Josef Medical Center, University of Regensburg, Landshuter Str. 65, 93053 Regensburg, Germany; 2 Institute of Pathology, Comprehensive Cancer Center EMN, University Hospital Erlangen, Friedrich-Alexander-Universität Erlangen-Nürnberg, Erlangen, Germany; 3 Department of Urology and Pediatric Urology, Friedrich-Alexander-University Erlangen-Nuremberg, Erlangen, Germany; 4 Department of Urology and Pediatric Urology, University Hospital Ulm, Ulm, Germany; 5 Department of Urology, Marien Hospital, Ruhr-University Bochum, Herne, Germany; 6 Stratifyer Molecular Pathology GmbH, Cologne, Germany; 7 Institute of Pathology, St. Elisabeth Hospital Köln-Hohenlind, Cologne, Germany; 8 Department of Urology and Urosurgery, University Hospital Mannheim Medical Faculty Mannheim, University of Heidelberg, Mannheim, Germany; 9 Department of Urology and Pediatric Urology, University Hospital Bonn, Bonn, Germany; 10 Biostatistics, Cepheid Inc., Sunnyvale, CA USA; 11 Medical and Scientific Affairs, Cepheid Inc., Sunnyvale, CA USA; 12 BRIDGE (Bladder Cancer Research Initiative for Drug Targets Germany) Consortium E.V., Mannheim, Germany

**Keywords:** Urological cancer, Cancer, Tumour biomarkers, Biomarkers, Diagnostic markers, Urology, Bladder

## Abstract

To determine whether Xpert bladder cancer monitor, a noninvasive PCR-based biomarker test, can predict the need for 2nd transurethral resection of the bladder (TURB) better than clinical assessment. Patients scheduled for TURB were prospectively screened. After initial TURB, patients were assigned to 2nd TURB or follow-up cystoscopy at 3 months (FU) by clinicians’ discretion. Central urine cytology and Xpert monitor tests were performed prior to the 1st TURB and 2nd TURB or FU, respectively. Statistical analysis to compare clinical assessment and Xpert monitor comprised sensitivity (SENS), specificity (SPEC), NPV and PPV. Of 756 screened patients, 171 were included (114 with 2nd TURB, 57 with FU). Residual tumors were detected in 34 patients who underwent 2nd TURB, and recurrent tumors were detected in 2 patients with FU. SENS and SPEC of Xpert monitor were 83.3% and 53.0%, respectively, PPV was 32.6% and NPV was 92.1%. Clinical risk assessment outperformed Xpert monitor. In patients with pTa disease at initial TURB, Xpert monitor revealed a NPV of 96%. Xpert monitor was not superior than clinical assessment in predicting the need for 2nd TURB. It might be an option to omit 2nd TURB for selected patients with pTa disease.

## Introduction

Seventy-five percent of newly diagnosed bladder cancers are non-muscle-invasive (NMIBCs) with a very good (~ 93%) five-year survival despite very high recurrence rates^1, 2^. The majority of patients with NMIBC are treated with organ-sparing transurethral resection of the bladder tumor (TURB)^3^. However, across different studies, the rate of residual tumor detected by a 2nd TURB varies between 27 and 78%. Furthermore, there is a significant risk of understaging the tumor: in patient with initial pT1 disease muscle-invasion is detected in 4% to 25% by the 2nd TURB^4–6^. Therefore, guidelines recommend a 2nd TURB within 2–6 weeks after initial resection in patients with risk factors^7,8^.

Although 2nd TURB is beneficial to some patients, this approach involves a relevant share of patients without tumor undergo surgery. This constitutes an unnecessary risk for perioperative complications, a hospital-stay and a delay in adjuvant therapy. Moreover, TURB is a major cost driver of bladder cancer treatment^9–11^.

Therefore, there is a need to better identify those patients who benefit from a 2nd TURB meanwhile decreasing unnecessary 2nd TURB. Since positive cytology is an independent prognostic factor of a residual tumor in the 2nd TURB^12^, residual tumors might also be detected by molecular urine markers with increased sensitivity for bladder tumors.

Xpert Bladder Cancer Monitor (Cepheid, Sunnyvale, CA) (Xpert Monitor) was developed and validated for monitoring the recurrence of NMIBC^13^. In a prospective, multicenter study the Xpert Monitor had an improved overall negative predictive value (NPV) compared to UroVysion and cytology. The overall sensitivity and specificity of Xpert Monitor to detect bladder cancer is 75% and 89.6%^14–16^.

The aim of this prospective multicenter study was to determine if employing the Xpert Monitor prior to 2nd TURB can reliably predict residual tumors and identify patients not requiring 2nd TURB.

## Materials and methods

### Objectives

The primary objective of this prospective, multicenter study was to determine if Xpert Monitor can predict the presence of residual tumor at 2nd TURB more accurately than clinical risk assessment.

Clinical recommendations for performing second TURB:

- Incomplete resection of the tumor during the initial TURB
- If there is no muscle in the specimen after initial resection, with the exception of TaG1 tumors and primary CIS
- In all T1 tumors
- In all HG/G3 tumors, except primary CIS

The indication for a 2nd TURB was determined by the clinical risk assessment of the treating physician at the specific center based on the German S3 Guidelines^8,9^.

2nd TURB was performed within 2–6 weeks after initial resection. A delayed 2nd TURB up to 12 weeks was permitted in select cases due to the COVID-19 pandemic.

In patients selected for monitor cystoscopy and not for 2nd TURB Xpert Monitor was performed at the time of cystoscopy and compared to endoscopic finding (for disease-negative patients) or histology (for disease-positive patients). The findings allowed for the determination of the specificity of the clinical assessment. Therefore, patients with a tumor detected at the first surveillance visit were considered ‘false negative by clinical assessment’.

### Study population

Patients (minimum 18 years of age) scheduled for TURB were prospectively screened and patients with NMIBC (first diagnosis or recurrent) scheduled for a 2nd TURB or follow-up cystoscopy were then enrolled. Patients with no malignancy, early cystectomy, MIBC or BCG therapy within 42 days before enrollment were excluded. Findings, data acquisition, and processing complied with the latest Declaration of Helsinki ethical standards. The study was approved by the University of Regensburg local ethics committee (Ethics vote: 18-967-101) and registered in the German Register of Clinical Trials (DRKS-ID: DRKS00014974). All patients gave written informed consent.

### Study procedures

Voided urine collection for both Xpert test and cytology evaluation was performed at the preintervention consultation 1–10 days before both the initial and second TURB (photodynamic diagnostic (PDD) was permitted) or follow-up cystoscopy. The urine sample was mixed with the Xpert Urine Transport Reagent within 1 h of collection and sent to a central lab (Institute of Pathology, Friedrich-Alexander-University of Erlangen-Nuremberg) within 3–5 days. Xpert Monitor performance was established relative to histology (for disease-positive and -negative patients) and cytology, performed at a central pathology lab (Institute of Pathology, Friedrich-Alexander-University of Erlangen-Nuremberg). Urine cytology was assessed according to the Paris system for reporting urinary cytology by two blinded expert pathologists (A.H., M.E.)^17^.

The results from Xpert Monitor were not used for patient management. To minimize bias in specimen analysis by Xpert Monitor, cytology, cystoscopy or TURB, the following procedures were employed:

- The operator(s) performing Xpert Monitor testing were blinded to patient status, cystoscopy, cytology and central histology results.
- The person(s) performing the TURB, cystoscopy and pathology analysis were blinded to the Xpert Monitor results.

### Xpert bladder cancer monitor (Xpert monitor)

Xpert Monitor, performed on the Cepheid GeneXpert Instrument Systems, is a qualitative in vitro diagnostic test intended to monitor for the recurrence of bladder cancer in patients previously diagnosed with bladder cancer. The test utilizes a voided urine specimen and measures the level of five target mRNAs (ABL1, CRH, IGF2, UPK1B, ANXA10) by means of real-time reverse transcription-polymerase chain reaction (RT–PCR).

Xpert Monitor provides POSITIVE or NEGATIVE test results based on the results of a proprietary linear discriminate analysis (LDA) algorithm that utilizes the cycle threshold (Ct) results of the five mRNA targets. It is not necessary to detect all of the mRNA targets for a POSITIVE test result. The predefined cut-off for a positive or negative result 0.5 was used in this study.

### Statistical analysis

The study was based on a superiority design to prove that Xpert Monitor alone performed more effectively than current clinical risk assessment to identify disease-positive patients (bladder cancer in pathology evaluation) with residual tumor after the first TURB. Xpert Monitor needed to be superior in specificity (= identify more accurately disease-negative patients who do not need a 2nd TURB) and equal to or better in sensitivity (= identify correctly disease-positive patients who need a 2nd TURB) than clinical assessment. Performance of clinical assessment was determined as sensitivity and specificity of prediction of residual tumor through clinical risk assessment as described in 2.1.

The comparison of the Xpert Monitor versus clinical assessment was carried out by McNemar Test. Success was achieved if the criterion *p* value < 0.05 was satisfied.

Statistical analysis was performed using SPSS version 26.0 (*IBM Deutschland GmbH, Ehningen, Germany*).

Sample size calculation was based on the assumption that 25% of all bladder cancer patients receive a second TURB where 30% had residual tumor. A minimum of 600 patients was calculated to achieve 150 patients who were selected for a second TURB based on clinical assessment; of these patients, 30–60 were anticipated to be disease-positive patients (= residual tumor detected in the 2nd TURB).

## Results

### Patient cohort

Between 01.07.2018 and 31.12.2020, a total of 756 patients were screened at the six urological university centers. Of those, 171 patients were included in the final analysis (Table 1).

**Table 1:** Clinical and histopathological characteristics at study inclusion at 1st TURB.

Parameter	*n* (%)
Patient data	
Total study cohort	171 (100)
First diagnosis	116 (68)
Male patients	139 (81)
Median age (years)	72 [IQR, 63–80]
Clinical and pathological parameters	
Staging	
pTa	115 (67)
pT1	53 (31)
pTis	3 (2)
Grading WHO1973	
G1	40 (23)
G2	69 (40)
G3	62 (36)
Grading WHO2004/2016	
Low-grade	82 (48)
High-grade	89 (52)
Tumor diameter	
< 30 mm	117 (68)
≥ 30 mm	39 (23)
n.a	15 (9)
Concomitant CIS	
Yes	22 (13)
Focality	
Multifocal	87 (51)
Muscle available	
Yes	131 (77)
EAU NMIBC risk group	
Low	17 (10)
Intermediate	78 (46)
High	60 (35)
Very high	16 (9)

**Figure 1:**
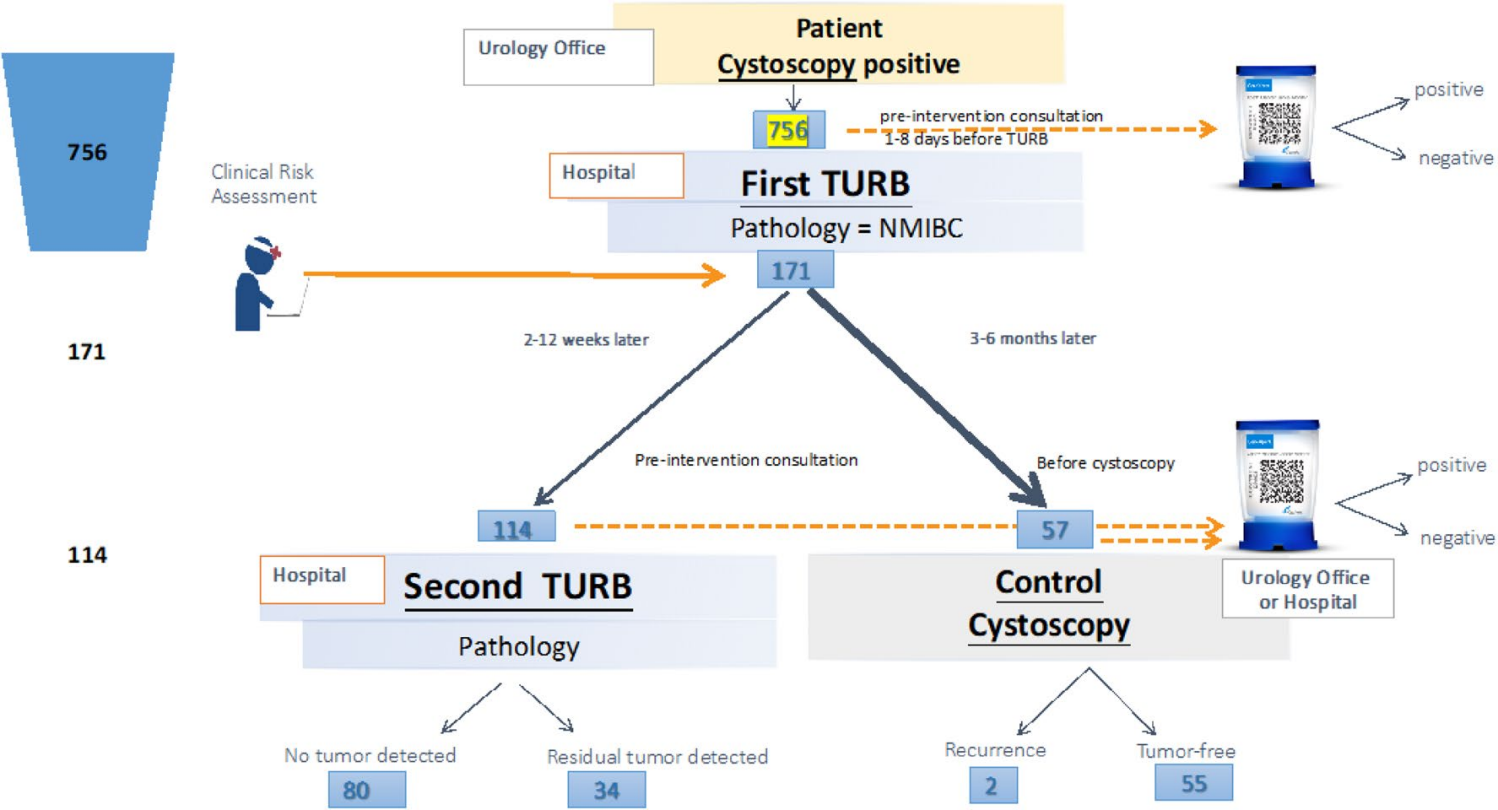
Flow-chart of study design, screened (n = 756) and included (n = 171) patients.

Patients were assigned to 2nd TURB in 114 (67%) cases (Fig. 1). Of those with 2nd TURB, 34 (30%) had a residual tumor, and none were upstaged to MIBC. Complete data, including urine analysis, were available for 57 patients who were assigned to the follow-up group by the physician’s assessment. Of those, 4 were referred to TURB after the follow-up visit with suspicion of recurrent tumor, and 2 of the 57 (4%) had a histologically proven recurrence (both pTa low-grade) (Table 2).

**Table 2:** Clinical and pathological parameters at second visit (2nd TURB or 3-month follow-up visit).

Parameter	2nd TURB (n = 114, 66.7%)	Follow-up (n = 57, 33.3%)
Histology		
No malignancy	80 (70)	2 (50)
Urothelial carcinoma	34 (30)	2 (50)
Focality		
Unifocal	15 (44)^a^	0 (0)^a^
Multifocal	14 (41)^a^	2 (100)^a^
n.a	5 (15)^a^	0 (0)^a^
Tumor diameter (largest)		
< 3 cm	22 (65)^a^	2 (100)^a^
≥ 3 cm	5 (15)^a^	0 (0)^a^
n.a	7 (20)^a^	0 (0)^a^
T-Stage		
pTa	17 (50)^a^	2 (100)^a^
pT1	10 (29)^a^	0 (0)^a^
pTis	7 (21)^a^	0 (0)^a^
MIBC	0 (0)^a^	0 (0)^a^
Concomitant CIS		
No	25 (74)^a^	0 (0)^a^
Yes	9 (26)^a^	0 (0)^a^
Grading1973		
G1	4 (12)^a^	2 (100)^a^
G2	8 (23)^a^	0 (0)^a^
G3	22 (65)^a^	0 (0)^a^
Grading2016		
Low grade	6 (18)^a^	2 (100)^a^
High grade	28 (82)^a^	0 (0)^a^

In Follow-up cystoscopy 4 of 57 patients (7%) had a suspicious finding and were referred to TURB.

^a^Numbers refer to cases with histologically proven urothelial carcinoma of the bladder.

### Performance of clinical assessment

Using clinical assessment, the sensitivity was 94%, and the specificity was 41% (Table 3a). The negative predictive value (NPV) of the clinical assessment was 97%, and the positive predictive value (PPV) was 30%, with an accuracy of 52% (Table 3a). The sensitivity for high-grade tumors in the 2nd resection or at the 3-month follow-up was 100% (28/28) and 75% for low-grade tumors (6/8) with two low-grade recurrences at the 3-month follow-up.

**Table 3:** Performance of clinical assessment to predict the need for 2nd TURB in the total cohort.

Group	n	TP	FP	TN	FN	Sensitivity	Specificity	HG	LG	PPV	**NPV**
(a) Clinical assessment by treating physician’s discretion											
Control cystoscopy	57	0	0	55	2	0/2 (0%)	55/55 (100%)		0/2 (0%)		55/57 (96.5%)
2nd TURB	114	34	80	0	0	34/34 (100%)	0/80 (0%)	28/28 (100%)	6/6 (100%)	34/114 (29.8%)	
Total	171	34	80	55	2	34/36 (94.4%)	55/135 (40.7%)	28/28 (100%)	6/8 (75%)	34/114 (29.8%)	55/57 (96.5%)
(b) Clinical assessment in pTa tumors by physician’s discretion											
Control cystoscopy	55	0	0	53	2	0/2 (0%)	53/53 (100%)		0/2 (100%)		53/55 (96.4%)
2nd TURB	60	15	45	0	0	15/15 (97.1%)	0/45 (0%)	11/11 (100%)	4/4 (66.7%)	15/60 (25%)	
Total	115	15	45	53	2	15/17 (88.2%)	53/98 (54.1%)	11/11 (100%)	4/6 (66.7%)	15/60 (25%)	53/55 (96.4%)

TP, true positive; FP, false positive; TN, true negative; FN, false negative; HG, sensitivity for high-grade; LG, sensitivity for low-grade; PPV, positive predictive value; NPV, negative predictive value.

In pTa tumors, the sensitivity for clinical assessment was 88%, and the specificity was 54%, NPV was 96% (Table 3b).

### Performance of Xpert monitor

Xpert Monitor revealed a positive result in 123 patients (72%) at the initial TURB; in 2 cases, repeat Xpert tests did not reveal a valid result and were determined to be “invalid” (Table 4). The sensitivity for Xpert Monitor at the initial TURB was 71% for all patients, 89% for high-grade tumors and 56% for low-grade tumors.

**Table 4:** Results of Xpert monitor and urine cytology per visit (n = 171).

	First visit (TURB)	Second visit (2nd TURB and follow-up)
Xpert monitor		
Negative	46 (26.9)	76 (44.4)
Positive	123 (71.9)	103 (53.8)
Invalid^a^	2 (1.2)	3 (1.8)
Mean LDA-value [IQR]	0.834 [0.461–1.166]	0.519 [0.377–0.701]
Urine cytology		
Paris classification		
Unsuitable probe	20 (11.7)	19 (11.1)
Atypical negative	14 (8.2)	10 (5.8)
Negative for high grade UCC	67 (39.2)	123 (71.9)
High grade UCC	69 (40.4)	19 (11.1)
n.a	1 (0.6)	0
Binary		
Negative	101 (59.1)	152 (88.9)
Positive	69 (40.4)	19 (11.1)
n.a	1 (0.6)	0

^a^Results were determined invalid, if both Xpert tests from the urine were invalid.

At the second visit (2nd TURB or follow-up), Xpert Monitor was positive in 103 patients (54%), and 3 cases were determined to be “invalid” (Table 4). When using Xpert Monitor to predict the need for a 2nd TURB, the sensitivity was 83%, and the specificity was 53%, with a NPV of 92% and a PPV of 33% (Table 5a). The sensitivity for high-grade tumors in the 2nd resection or at the 3-month follow-up was 86% (24/28) and 75% for low-grade tumors (6/8). Using Xpert monitor alone, 40 2nd TURBs (36%) could have been omitted at the cost of 4 missed high-grade tumors (only 1 of these 4 had negative Xpert results at both visits, the other 3 had a positive result at the initial visit). McNemar test comparing Xpert results and clinical assessment revealed that Xpert test was not different to clinical assessment, *p* = 0.063.

**Table 5:** Performance of Xpert monitor to predict the need for 2nd TURB.

Group	n	TP	FP	TN	FN	Sensitivity	Specificity	HG	LG	PPV	NPV
(a) Total cohort (n = 171)											
Control cystoscopy	56	2	18	36	0	2/2 (100%)	36/54 (66.7%)		2/2 (100%)	2/20 (10%)	36/36 (100%)
2nd TURB	112	28	44	34	6	28/34 (82.4%)	34/78 (43.6%)	24/28 (85.7%)	4/6 (66.7%)	28/72 (38.9%)	34/40 (85.0%)
Total	168	30	62	70	6	30/36 (83.3%)	70/132 (53.0%)	24/28 (85.7%)	6/8 (75%)	30/92 (32.6%)	70/76 (92.1%)
(b) Patients with muscle in specimen at initial TURB (n = 131)											
Control cystoscopy	36	1	15	20	0	1/1 (100%)	20/35 (57.1%)		1/1 (100%)	1/16 (6.3%)	20/20 (100%)
2nd TURB	93	24	37	28	4	24/28 (85.7%)	28/65 (43.1%)	20/24 (83.3%)	4/4 (100%)	24/61 (39.3%)	28/32 (87.5%)
Total	129	25	52	48	4	25/29 (86.2%)	48/100 (48%)	20/24 (83.3%)	5/5 (100%)	25/77 (32.5%)	48/52 (92.3%)
(c) Patients with pTa tumor at initial TURB (n = 115)											
Control cystoscopy	54	2	16	36	0	2/2 (100%)	36/52 (69.2%)		2/2 (100%)	2/18 (11.1%)	36/36 (100%)
Second TURB	59	14	25	19	1	14/15 (93.3%)	19/44 (43.2%)	11/11 (100%)	3/4 (66.7%)	14/39 (35.9%)	19/20 (95.0%)
Total	113	16	41	55	1	16/17 (83.3%)	55/96 (57.3%)	11/11 (100%)	5/6 (83.3%)	16/57 (28.1%)	55/56 (98.2%)

TP, true positive; FP, false positive; TN, true negative; FN, false negative; HG, sensitivity for high-grade; LG, sensitivity for low-grade; PPV, positive predictive value; NPV, negative predictive value.

During the initial resection, 131 patients had muscle in the specimen. When using Xpert Monitor in these 131 patients with muscle specimens to predict the need for a 2nd TURB, the sensitivity was 86%, the specificity was 48%, the NPV was 92% and the PPV was 33% (Table 5b).

When using Xpert monitor in the 115 patients with a pTa tumor at initial resection (67% of the included patients) to predict the need for a 2nd TURB, the sensitivity was 83%, specificity 57%, NPV 98% and PPV 28% (Table 5c). The sensitivity for high-grade tumors in the 2nd resection or at the 3-month follow-up was 100% (11/11) and 83% for low-grade tumors (5/6).

Looking at EAU NMIBC risk groups, almost all low-risk patients (94%) underwent follow-up, whereas almost all high-risk patients (90%) and all very high-risk patients underwent re-TUR (Suppl. Table S1). Compared to clinical assessment, especially in intermediate risk patients, Xpert outperformed clinical assessment (sensitivity: 90% vs. 80%; specificity: 54% vs. 52%; PPV: 23% vs. 20%; NPV: 97% vs. 95%). Xpert monitor was not better than clinical assessment in the other risk groups (Suppl. Table S1).

### Performance of urine cytology

Urine cytology was positive in 69 patients (40%) at the initial TURB; in 1 case, urine was not sent for central evaluation (Table 4). The sensitivity for urine cytology at the initial TURB was 59% for all patients, 60% for high-grade tumors and 20% for low-grade tumors.

When using urine cytology to predict the need for a 2nd TURB, the sensitivity was 33%, and the specificity was 95%, NPV was 84% and PPV 63%, respectively (Table 6a).

**Table 6:** Performance of Urine cytology to predict the need for 2nd TURB.

Group	n	TP	FP	TN	FN	Sensitivity	Specificity	HG	LG	PPV	NPV
(a) Total cohort (n = 171)											
Control cystoscopy	57	1	3	52	1	1/2 (50%)	52/55 (94.5%)		1/2 (50%)	1/4 (25%)	52/53 (98.1%)
2nd TURB	114	11	4	76	23	11/34 (32.4%)	76/80 (95.0%)	9/28 (32.1%)	2/6 (33.3%)	11/15 (73.3%)	76/99 (77.8%)
Total	171	12	7	128	24	12/36 (33.3%)	128/135 (94.8%)	9/28 (32.1%)	3/8 (37.5%)	12/19 (63.2%)	128/152 (84.2%)
(b) Combination of Xpert monitor and urine cytology (neg = Xpert neg AND Cytology neg; pos = Xpert pos AND / OR Cytology pos) in the total cohort (n = 171)											
Control cystoscopy	57	2	19	36	0	2/2 (100%)	36/55 (65.5%)		2/2 (100%)	2/21 (9.5%)	36/36 (100%)
2nd TURB	114	29	45	35	5	29/34 (85.3%)	35/80 (43.8%)	27/28 (96.4%)	5/6 (83.3%)	29/74 (39.2%)	35/40 (87.5%)
Total	171	31	64	71	5	31/36 (86.1%)	71/135 (52.6%)	27/28 (96.4%)	7/8 (87.5%)	31/95 (32.6%)	71/76 (93.4%)

TP, true positive; FP, false positive; TN, true negative; FN, false negative; HG, high-grade; LG, low-grade; PPV, positive predictive value; NPV, negative predictive value.

When combining Xpert monitor and urine cytology results to predict the need for 2nd TURB, the sensitivity was 86%, specificity 53%, NPV 93% and PPV 33% (Table 6b).

## Discussion

In this multicenter trial, we prospectively assessed a large cohort of patients undergoing first and second TURB. Among our patients scheduled for a second TURB, 30% had residual tumor. Hence, tumor persistency in our cohort was lower than in previous studies. Persistent disease after resection of T1 tumors was observed in 33–55% of patients and 41.4% after resection of TaG3 tumors^18,19^. The lower incidence of residual tumor in our cohort may be explained by the frequent use of PDD in all centers, which has recently been shown to almost halve tumor persistency at second TURB^20^. Another explanation may be the rather liberal indication to perform a second TURB by the physicians involved in this study. The German S3 Guidelines recommend second TURB in any high-grade tumor, increasing the likelihood for patients to schedule a second TURB^8^.

The majority of the patients undergoing second TURB have no evidence of disease, and second TURB is overtreatment. It constitutes a risk of perioperative complications, a decrease in quality of life and a delay in adjuvant instillation therapy^5^. The overall complication rate of TURB has been shown to be approximately 20%, with 2–3% of patients suffering from major complications^9,21^. Prospective assessment of quality of life shows that following TURB, more than half of patients suffer from substantial voiding problems. Reduced sexual function, anxiety and depression have also been reported by a significant proportion of patients^11,22,23^. Therefore, urologists should seek to reduce the number of second TURBs without compromising oncology effects.

In addition to the established clinical risk factors, parameters such as the number of T1 chips, ploidy of tumors, employment of PDD or en bloc resection have been evaluated as predictors of residual tumor without gaining broad clinical significance^3,12,20,24^. Urinary biomarkers have shown some value in the detection of bladder cancer. A recent single center study examined the Xpert Monitor as a predictor of residual tumor at the second TURB^25^. The authors found a sensitivity of 85.9% and a specificity of 72.3% (95% CI 68–76) for the detection of tumors at repeat TURB. In this single center cohort of 245 patients with resected T1 disease, there were 14 patients with false negative results, all revealing G3/HG tumors at the second TURB.

In our prospective multicenter study, patients with any indication (in addition to pT1 disease) for a second TURB or surveillance were included. We found a sensitivity of 83.3% and a specificity of 53.0% for the prediction of residual tumor. Thus, the study did not meet the primary endpoint. A reason for the lower performance of the Xpert Monitor in this study may be explained by the lower prevalence of residual tumor in our sample, since we included pTa tumors undergoing second TURB and a control arm of patients under surveillance. The number of invalid results of the Xpert Monitor was low at 1.5% and within the range of previous studies^14^.

Overall, 6 patients had false negative results according to the Xpert Monitor prior to the second TURB, including 4 patients with high grade tumors and 2 patients with pT1 tumors. All of them had pT1 disease in first resection. If we had been relying solely on the urinary biomarker, we would have spared 40 TURBs (36%) at the cost of overseeing 4 patients with high grade tumors. The subsequent impact on the oncology outcomes of these four patients remain unclear. One could hypothesize that an early start of adjuvant treatment and a control cystoscopy at 3 months would strongly limit the potential of these malignancies to upstage and progress rapidly without recognition. Admittedly, the effect of a second TURB on recurrence and survival has recently been questioned by a large retrospective study and a recent meta-analysis^22,26^. On the other hand, sparing 36% of most likely unnecessary second TURBs would mean a significant benefit not only to the respective patients but also to health care systems^10,27^. The costs of the Xpert Monitor add up to approximately 150€ compared to 2100€ for TURB.

However, there is still a risk for upstaging in second TURB that impacts the oncological prognosis of the patient dramatically^28^. In this study, we did not find any upstaging to pT2. This may be explained by the relatively low share of pT1 tumors at initial TURB and the quality of tumor resection. Another explanation may be the fact that 14 of the 756 screened patients (1.9%) were assigned to immediate cystectomy for NMIBC and did not undergo second TURB. In cohorts of pure pT1 disease, studies show a risk of upstaging of up to 20%^3,5,28^. Therefore, physicians will always require the excellent performance of biomarkers and predictors to avoid omitting a second TURB. Furthermore, the data on the long-term oncological benefit of second TURB are strongest in pT1 patients^18^.

According to the German S3 guidelines, patients with pTa high-grade tumors should undergo a second TURB, according to the EAU guidelines in case there is no detrusor in the specimen^7,8^. In the post hoc subgroup analysis, the Xpert monitor performed well with a sensitivity of 83% and a NPV of 98%. Indeed, the Xpert monitor missed zero high-grade tumors and only one low-grade tumor at the second TURB. Remarkably, it could have spared 34% of second TURBs in this group of patients. These findings, in combination with the fact that many bladder cancer patients are elderly and have significant comorbidities, demonstrate a potential field of application for the Xpert Monitor prior to second TURB.

This is also reflected by an improved sensitivity, specificity, PPV and NPV in intermediate-risk tumors, which mainly consist of pTa tumors. As described above, almost all low-risk did undergo follow-up, whereas almost all high-risk and all very high-risk patients underwent 2nd TURB and Xpert monitor was not better than clinical assessment in these patients.

Despite its prospective multicenter design and central pathological evaluation this study is not without limitations. First the share of patients with pT1 disease in this cohort is only 31% leading to a general low risk of tumor persistency at second TUR-B. Secondly, the clinical decision to perform second TUR-B was also depended on the surgeon’s assessment and thus not standardized over the different centers. Finally, the study has no oncological long-term follow-up, which however is not relevant for the study’s primary endpoint.

## Conclusion

In this prospective multicenter biomarker trial, tumor persistency at second TURB was found in 30% of the patients. The Xpert Monitor test revealed a sensitivity of 83% and a negative predictive value of 92% in predicting residual tumor at second TURB. Its clinical application could spare more than one-third of second TURBs at the cost of missing 14% of high-grade tumor persistency. In the large subgroup of pTa tumors (67%), the Xpert Monitor test performed well with a negative predictive value of 98% and would have spared 34% of 2nd TURBs. Therefore, the Xpert Monitor could be useful in sparing second TURB in elderly and comorbid patients with pTa disease.

### Supplementary Information

Supplementary Table S1.

## Data Availability

The data sets generated during and/or analyzed during the current study are available from the corresponding author on reasonable request.
