# Supplementary material for: Xpert bladder cancer monitor to predict the need for a second TURB (MoniTURB trial)
Source: Sci Rep. 2023 Sep 18;13:15437. doi: 10.1038/s41598-023-42088-z (PMC10507065; doi:10.1038/s41598-023-42088-z)
Supplement: Supplementary file 1 — Supplementary Table S1. [file 41598_2023_42088_MOESM1_ESM.docx]

**Suppl. Table 1.** Performance of clinical assessment, Xpert monitor and cytology according to EAU risk groups.

| **EAU risk group** |  | **Clinical decision** | **Xpert test** | **cytology** |
| --- | --- | --- | --- | --- |
| **Low risk**  *17 patients*  *16 follow-up*  *1 re-TUR*  *No residual tumor* | Sens |  |  |  |
|  | Spec | 16/17  (94.1%) | 14/16  (87.5%) | 17/17  (100%) |
|  | PPV |  |  |  |
|  | NPV | 16/16  (100%) | 14/14  (100%) | 17/17  (100%) |
| **Intermediate risk**  *78 patients*  *37 follow-up*  *41 re-TUR*  *10 residual tumors* | Sens | 8/10  (80%) | 9/10  (90%) | 4/10  (40%) |
|  | Spec | 35/68  (51.5%) | 37/68  (54.4%) | 65/68  (95.6%) |
|  | PPV | 8/41  (19.5%) | 9/40  (22.5%) | 4/7  (57.1%) |
|  | NPV | 35/37  (94.6%) | 37/38  (97.4%) | 65/71  (91.5%) |
| **High risk**  *60 patients*  *4 follow-up*  *54 re-TUR*  *19 residual tumors* | Sens | 19/19  (100%) | 15/19  (78.9%) | 5/19  (26.3%) |
|  | Spec | 4/41  (9.8%) | 15/39  (38.5%) | 37/41  (90.2%) |
|  | PPV | 19/56  (33.9%) | 15/39  (38.5%) | 5/9  (55.6%) |
|  | NPV | 4/4  (100%) | 15/19  (78.9%) | 37/51  (72.5%) |
| **Very high risk**  *16 patients*  *all re-TUR*  *7 residual tumors* | Sens | 7/7  (100%) | 7/7  (100%) | 4/7  (57.1%) |
|  | Spec |  | 2/9  (22.2%) | 2/9  (22.2%) |
|  | PPV | 7/16  (43.8%) | 7/14  (50%) | 4/11  (36.4%) |
|  | NPV |  | 2/2  (100%) | 2/5  (40%) |
